# The Gold Standard Publication Checklist (GSPC) for improved design, reporting and scientific quality of animal studies GSPC versus ARRIVE guidelines

**DOI:** 10.1258/la.2010.010130

**Published:** 2011-01

**Authors:** C Hooijmans, R de Vries, M Leenaars, M Ritskes-Hoitinga

**Affiliations:** Radboud University Nijmegen Medical Centre – Central Animal Laboratory, 3R Research Centre, Geert Grooteplein Noord 29 route 231, Nijmegen 6525 EZ, The Netherlands

Hundreds of experiments in which animals are used to answer biomedical research questions are performed and published every month. Although well-designed and performed animal experiments are a necessary condition for successful translational research, many papers involving animal experimentation are still incomplete in their reporting.^1–4^ Clearly, there is an urgent need to improve the reporting of animal experiments in order to increase the scientific quality of animal studies, animal welfare and ultimately patient safety.^5–7^ Currently, the paradoxical situation exists that the high standards set for clinical trials are not applied in animal experiments, even though these animal studies are performed with the aim to improve human health care. Against this background, the ARRIVE guidelines were published in *PLoS Biology* in July 2010.^8^ We strongly support this initiative and believe it will make an important contribution to improving the reporting of animal studies.

In our view, guidelines are not only necessary for increasing the quality of reporting of completed animal studies, but are also essential for optimal design and execution of new animal experiments, and thus improved scientific quality. With these goals in mind, we developed the Gold Standard Publication Checklist (GSPC): Hooijmans *et al.* A gold standard publication checklist to improve the quality of animal studies, to fully integrate the three Rs, and to make systematic reviews more feasible. *Altern Lab Anim* 2010;**38**(2):167–82. This checklist was presented and discussed at the World Congress on alternatives and animal use in the life sciences in Rome in 2009 and published in *ATLA* in May 2010, a few months before the ARRIVE guidelines appeared.

Given their partly similar aims, the GSPC has some overlap with the ARRIVE guidelines. However, the GSPC describes certain items in more detail, for instance the housing conditions (humidity, ventilation, lighting, noise, caging), nutrition (type of diet, diet content, method of feeding) and water. These detailed descriptions in the checklist help scientists to include all the specific items necessary for planning, designing and performing animal experiments in the most optimal way, and to improve repeatability of and control variation within experiments, through which the quality of research improves and the number of animals needed in an experiment diminishes. In addition, the GSPC paper highlights the importance of reporting husbandry conditions and basic principles of the design of animal experiments by providing an overview of the literature on how and when interference with experimental results may occur. Last but not least, the GSPC is presented as a checklist, and therefore well-ordered and easy to use when designing and executing animal experiments.

The use of guidelines for designing, executing and reporting of animal experiments (like the ARRIVE guidelines or the GSPC) will also make systematic reviews (SRs) and meta-analyses of publications on animal studies more feasible.^9,10^ SRs can be defined as a literature review focused on a single question that tries to identify, appraise, select and synthesize all available high-quality research evidence relevant to that question. These SRs lead to better interpretation of the already existing scientific results from animal experiments, through which a better translation to the clinic and more guarantees for patient safety become reality. Furthermore, unnecessary duplication of animal experiments, and thereby unnecessary animal use and time loss, will be prevented. SRs are already standard practice in clinical studies and it is about time that they will become standard practice in the field of animal studies as well.^3,11^

To conclude, in order to make SRs feasible and to improve not only the reporting but also the planning, design and execution of animal studies, we strongly recommend all scientists involved in animal experimentation and editors of journals publishing animal studies to make use of the GSPC and/or the ARRIVE guidelines.
